# Policy dismantling as evidence-based practice

**DOI:** 10.1186/1748-5908-10-S1-A47

**Published:** 2015-08-20

**Authors:** Neelum Arya

**Affiliations:** 1 University of California, Los Angeles School of Law, UCLA School of Law, Los Angeles, CA 90095-1476, USA

## 

Problems to be addressed: Every year approximately 100,000 youths under the age of 18 are admitted to adult jails across the country. These youths are in extreme danger. Many children are placed in solitary confinement which can cause anxiety, paranoia, and exacerbate existing mental disorders putting youths at increased risk of suicide. Youths housed in adult jails are 36 times more likely to commit suicide than are youths housed in juvenile detention facilities. Youths are also at great risk of sexual victimization. Even if youths are not physically or mentally harmed during their incarceration, youths are frequently denied education, exercise, and other programming, which compromises their overall development. Numerous studies and reports have documented problems inherent in housing youths in adult facilities, yet no study has specifically examined strategies used by counties to avoid housing youths in adult jails. I propose to present the initial findings and conceptual approach of this new research project.

## Research methods

The purpose of the Jail Removal Project at the UCLA School of Law is to assist local governments in removing youths from adult jails by identifying, documenting, and promoting strategies currently used by urban, suburban, and rural jurisdictions to house youths in appropriate juvenile settings. Mixed-methods research design using a combination of legal analysis; quantitative analyses of Bureau of Justice Statistics data; qualitative open-ended surveys of juvenile justice practitioners; case studies using site visits, interviews, and focus groups; and expert interviews.

## Advancing the field of D&I

The Jail Removal Project provides a new conceptual framework aimed at reconciling diverse literatures (policy implementation, diffusion, transfer, innovation diffusion, and legal mobilization). Existing studies have not adequately conceptualized the need for policy dismantling to advance public health. The Jail Removal Project provides a new conceptual framework aimed at reconciling these diverse literatures.

## Source of funding

Public Welfare Foundation.

